# The Video Head Impulse Test (vHIT) Detects Vertical Semicircular Canal Dysfunction

**DOI:** 10.1371/journal.pone.0061488

**Published:** 2013-04-22

**Authors:** Hamish Gavin MacDougall, Leigh Andrew McGarvie, Gabor Michael Halmagyi, Ian Stewart Curthoys, Konrad Peter Weber

**Affiliations:** 1 Vestibular Research Laboratory, School of Psychology, University of Sydney, Sydney, Australia; 2 Department of Neurology, Royal Prince Alfred Hospital, Sydney, Australia; 3 Department of Ophthalmology, University Hospital Zurich, Zurich, Switzerland; 4 Department of Neurology, University Hospital Zurich, Zurich, Switzerland; University of Iowa, United States of America

## Abstract

**Background:**

The video head impulse test (vHIT) is a useful clinical tool to detect semicircular canal dysfunction. However vHIT has hitherto been limited to measurement of horizontal canals, while scleral search coils have been the only accepted method to measure head impulses in vertical canals. The goal of this study was to determine whether vHIT can detect vertical semicircular canal dysfunction as identified by scleral search coil recordings.

**Methods:**

Small unpredictable head rotations were delivered by hand diagonally in the plane of the vertical semicircular canals while gaze was directed along the same plane. The planes were oriented along the left-anterior-right-posterior (LARP) canals and right-anterior-left-posterior (RALP) canals. Eye movements were recorded simultaneously in 2D with vHIT (250 Hz) and in 3D with search coils (1000 Hz). Twelve patients with unilateral, bilateral and individual semicircular canal dysfunction were tested and compared to seven normal subjects.

**Results:**

Simultaneous video and search coil recordings were closely comparable. Mean VOR gain difference measured with vHIT and search coils was 0.05 (SD = 0.14) for the LARP plane and −0.04 (SD = 0.14) for the RALP plane. The coefficient of determination *R^2^* was 0.98 for the LARP plane and 0.98 for the RALP plane and the results of the two methods were not significantly different. vHIT and search coil measures displayed comparable patterns of covert and overt catch-up saccades.

**Conclusions:**

vHIT detects dysfunction of individual vertical semicircular canals in vestibular patients as accurately as scleral search coils. Unlike search coils, vHIT is non-invasive, easy to use and hence practical in clinics.

## Introduction

Measures of the function of individual semicircular canals are invaluable for diagnosing peripheral vestibular loss, such as superior or inferior vestibular neuritis [1], [2]. At present measurement of semicircular canal function relies on measures of horizontal semicircular canal responses to caloric or rotational stimulation. At the bedside, the eye movement response to brief, unpredictable, passive, horizontal head rotations (called head impulses) is an important clinical indicator of the functional state of the horizontal semicircular canals [3]. A recent study provided objective measures of this response for the horizontal semicircular canals using high speed video measures of the eye movement response to head impulses - the video head impulse test (vHIT) and the quality of this test of horizontal canal function was validated by direct comparison with simultaneously recorded scleral search coil recordings [4]. In a preliminary study, we demonstrated that vHIT can also be used to detect vertical canal dysfunction [5]. Here we set out to validate the vHIT measures of vertical semicircular canals with simultaneous scleral search coil recordings (the current gold standard [6]) of the responses of subjects and patients, using patients with known peripheral vestibular losses.

**Figure 1:**
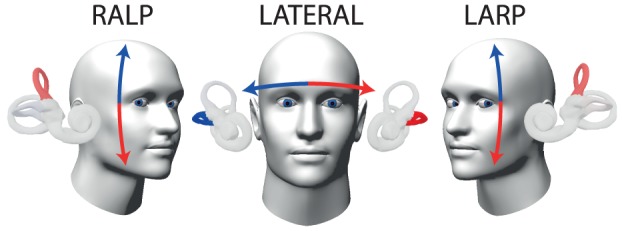
Modified head impulse procedure for vertical semicircular canals. Head impulses for RALP (right anterior – left posterior), LARP (left anterior – right posterior) and lateral canal stimulation (arrows), as viewed from the fixation point. For testing the vertical canals, a modified procedure has been used, which elicits mainly vertical eye movements to dispense with complex video processing of torsional eye movements [7]: The person's head is positioned turned with respect to the body, so that gaze is directed along the plane of head rotation in the direction of the named canals as represented by the vertical arrows. For testing horizontal canals the movement is in the plane of the horizontal canals as shown. These images are modified from the free iPhone or iPad app ‘aVOR’ developed by the first author [19]. For the examination procedure, see also accompanying Video S1.

To stimulate the vertical semicircular canals we used diagonal head impulses in the plane of the vertical semicircular canals (Figure 1, Video S1), while the head was turned and gaze was directed along the plane of head rotation [7]. The advantage of this modified head impulse procedure is that it elicits mainly vertical eye movements, so that two-dimensional measurements with video pupil tracking are sufficient for analysis and it is also much less uncomfortable for the subject than impulses in the plane of the vertical canals with the head aligned with the body midline.

## Materials and Methods

### Ethics statement

Written informed consent was obtained from all subjects and patients. The protocol was approved by the Sydney South West Area Health Service Ethics Committee in accordance with the Declaration of Helsinki.

### Subjects

19 subjects and patients were recorded simultaneously with video-oculography and scleral search coils. 7 healthy subjects without any history, symptoms, or clinical signs of vestibular disease (mean 35 years, age range 25–66 years, 2 females) served as controls. Patients with a wide range of previously identified vestibular deficits were enrolled: 5 patients with unilateral vestibular deafferentation after surgery for vestibular Schwannoma, 2 patients with bilateral vestibular loss (one idiopathic and one due to previous systemic gentamicin vestibulotoxicity), 2 patients after surgical canal occlusion for intractable benign paroxysmal positional vertigo (one bilateral posterior, one lateral), 2 patients with superior semicircular canal dehiscence (one operated, one un-operated) and 1 patient with an idiopathic, isolated posterior canal loss identified by prior testing with search coils. Patients were tested between 5 months and 27 years after onset of symptoms. Eligible patients were recruited at the Hearing and Balance Clinic, Royal Prince Alfred Hospital, Sydney, Australia. No potential subject was excluded. All subjects and patients were tested between March and November 2011.

### Study design

The study was a prospective, cross-sectional comparison of the head impulse test using video (vHIT) and scleral search coil measures of vertical canal function in patients with prior, independently identified vestibular deficits due to natural dysfunction or surgical blockage of the semicircular canals and healthy asymptomatic control subjects. Each person had all 6 semicircular canals tested.

### Experimental procedure

Subjects were instructed to fixate a laser dot on a screen at about 1 m distance in a dimly lit room. About 20 head impulses in the planes of the vertical canals were manually delivered by the experimenter with unpredictable timing and direction. Peak head velocity of the impulses ranged from 50 to 250°/s (acceleration 750–5000°/s^2^, amplitude 5–20°). The same (right) eye was recorded *simultaneously* with video-oculography and scleral search coils [4]. All recordings were performed by four of the co-authors (HGM, LAM, GMH, KPW) who were unmasked as to whether they were testing a patient or a healthy subject. All experimenters are university graduates with at least 5 years experience in vestibular research. There were no adverse events from performing the tests.

To test the anterior and posterior semicircular canals, the head rotations were delivered in the planes of the vertical canals – left anterior-right posterior (LARP) and right anterior-left posterior (RALP). These canals lie in planes, which are approximately 45° to the sagittal plane of the head [8]. Traditional head impulses in these diagonal planes, while gaze is directed straight ahead, elicit torsional and vertical eye movements, which require tree-dimensional analysis. To permit the use of only two-dimensional eye movement measures with video pupil tracking, we used the following modified procedure with gaze directed along the plane of head rotation, which elicits mainly vertical eye movements [7]. The person's head was positioned about 30–40° turned to the left or right with respect to their body so that the targeted vertical canal plane was approximately aligned with the body sagittal plane, then diagonal head movements were delivered in the plane of the vertical canals, while gaze was directed eccentrically along the plane of head rotation (Figure 1, Video S1). The experimenter placed one hand on top of the head, the other beneath the chin and for each impulse, moved the head abruptly through a small angle up or down (about 10–20°) with unpredictable direction and timing, while the person attempted to maintain fixation on the earth-fixed target. The direction of the head movement determines which canal of the pair is activated: so for example with the LARP pair - chin down activates the left anterior canal. In this way it is possible to selectively activate each vertical canal. Head impulses in the horizontal plane were also recorded to provide complete characterization of peripheral semicircular canal function.

### Video-oculography

The position of the right eye was recorded in two dimensions at 250 Hz with a small, lightweight high-speed digital (IEEE 1394a) video camera (Firefly MV, Point Grey Research Inc., Vancouver, BC) mounted on a lightweight frame that locked comfortably onto the bridge of the nose and around the eye sockets to minimize slippage of the camera relative to the head [4], [9]. The eye was illuminated by two infrared light emitting diodes (TSUS502, Vishay Intertechnology, Malvern, PA) that could be adjusted to gain optimum illumination of the eye. The image of the eye was reflected from an infrared mirror to the camera. Head velocity was measured by three miniature orthogonal gyroscopes (IDG-300, InvenSense, Santa Clara, CA) on the goggle frame and aligned with the canal planes – horizontal, LARP, and RALP. The light weight of the custom-built, non-commercial system (∼60 g) minimized inertia during head rotation and so minimized slippage of the glasses. Eye position was calibrated *in vivo* by asking patients to fixate projected targets from glasses-mounted miniature lasers. For the vertical impulses, the dots were separated 15° vertically at a position 35° left or 35° right gaze, and for the horizontal plane, the dots were separated by 15° horizontally. Video images were analysed online by a laptop to calculate two-dimensional eye position using pupil-tracking algorithms written in LabVIEW (National Instruments, Austin, TX). Eye velocity was obtained from a two-point differentiator and low-pass filtered (0–30 Hz bandwidth).

### Scleral search coil recording

Right eye and head position were recorded simultaneously to the video recording with the scleral search coil technique in a 1.9×1.9×1.9 m magnetic coil frame (CNC Engineering, Seattle, WA). Dual scleral search coils (Skalar, Delft, The Netherlands) were calibrated *in vitro* beforehand using a gimbal. The right eye was anesthetized topically with Alcaine 0.5% eye drops (Alcon Laboratories Australia Pty Ltd) and the eye coil inserted. The head coil was attached to a dental impression tray. Three-dimensional head and gaze position signals were low-pass filtered (0–100 Hz bandwidth), sampled at 1000 Hz and digitized with 16-bit precision. Three-dimensional rotation vectors and angular velocity vectors of head, gaze and eye were derived from coil voltages.

### Data analysis

For the simultaneous vHIT and search coil measures, offline analysis of the experimental data was carried out with customized LabVIEW software. Video and search-coil measurements were synchronized with a square-wave signal acquired by both recording systems. For the video measures, signals of vertical eye velocity (at eccentric eye position) and head velocity in the LARP or RALP plane were used. For the search coil measures, signals of vertical eye velocity (without torsion) and head velocity in the LARP or RALP plane were used. Head impulses were automatically detected and aligned at peak head acceleration. Trials with blinks and outliers were detected automatically based on an envelope around the expected eye velocity response and excluded from both recordings. Saccades were detected using an eye acceleration algorithm.

The gain of the VOR was calculated for both recording methods as the ratio of cumulative slow-phase eye velocity over cumulative head velocity from the onset of the head impulse to the moment when head velocity returned to zero. The points of these boundaries were defined from 60 ms before peak head acceleration to the last value of 0°/s as the head returned to rest. The same boundaries were applied to the desaccaded eye velocity response. The ratio of the area under the eye-velocity curve to the area under the head-velocity curve between these boundaries was used to calculate VOR gain. This method essentially corresponds to a desaccaded position gain. The new gain algorithm was preferred to the traditional method of calculating VOR gain using a few data points at around peak head velocity [10] because it is less susceptible to vHIT artefacts.

### Statistical analysis

Paired sample t-tests were used to test whether the VOR gains were different in video and search-coil recordings [11]. Due to the small sample size, no subgroup analyses were performed.

## Results

**Figure 2:**
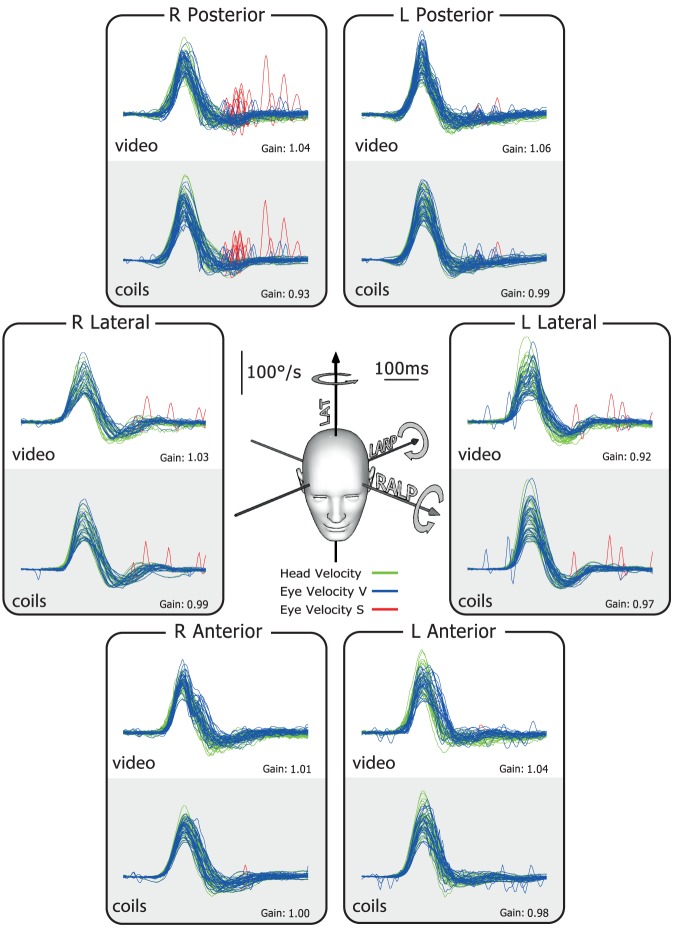
Simultaneous video and search coil head impulse recordings of all semicircular canals in a healthy subject. The six double panels in each figure show simultaneous measures by scleral search coils (lower half of each panel) and the video head impulse system (upper half of each panel). The records here show that for a healthy subject the traces of head and eye velocity are almost superimposed for the direction of each semicircular canal. The VOR gain values for head turns in every canal plane are in the normal range. The plots themselves in this and the following figures are time series showing superimposed records of the head velocity stimulus (head velocity – green traces) and the slow-phase eye-velocity responses (eye velocity VOR – blue traces) to about 20 brief unpredictable head turns in the direction of each semicircular canal. Overt or covert saccades are shown as red traces [10]. Tiny overt catch-up saccades are normal in healthy subjects. In these figures eye velocity has been inverted to allow easy comparison with head velocity, and for purposes of illustration both leftward and rightward head movements are shown as positive. The average VOR gain value is shown next to each group of responses. The inset at the centre shows the rotation axes of the semicircular canals being tested.

**Figure 3:**
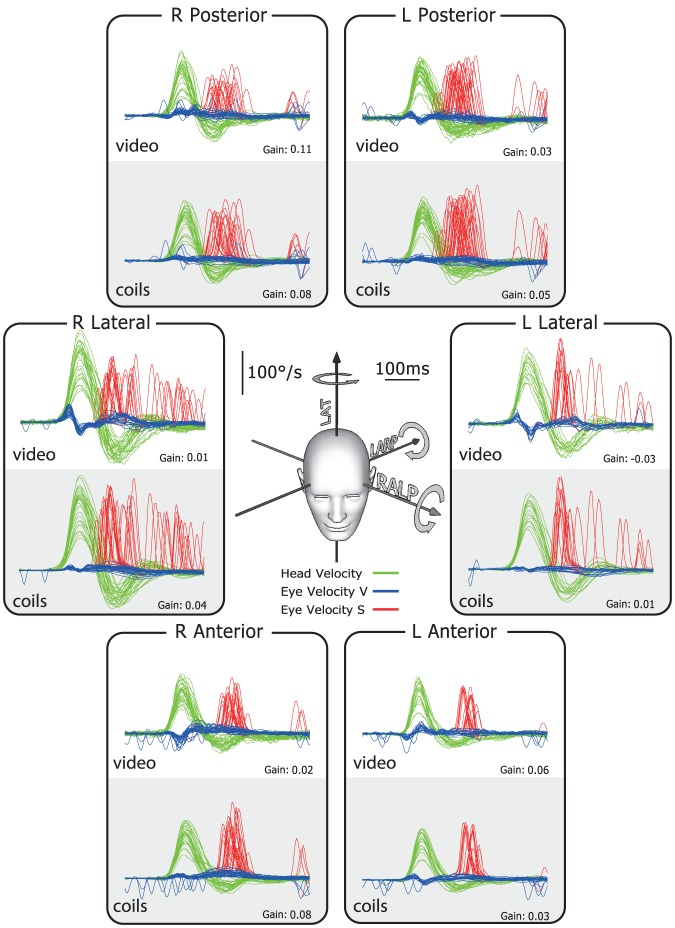
Simultaneous video and search coil head impulse recordings of all semicircular canals in a patient with idiopathic bilateral vestibular loss. For every direction of head rotation (green traces), there is no effective compensatory slow-phase eye velocity response (blue traces). There is a shower of saccades at the end of each head rotation (red traces). The VOR gain for all canals is close to zero.

The typical results for a healthy subject are shown in Figure 2. In this and the later figures, the plots show superimposed records of desaccaded eye-velocity responses (blue traces) to head-velocity stimuli (green traces) for about 20 brief, unpredictable, passive head rotations in each canal plane. Eye velocity has been inverted in these figures to allow easy comparison of eye velocity and head velocity. Saccades are shown in red. For both video and search coils the head velocity traces and eye velocity traces are almost superimposed for tests of each semicircular canal and the corresponding VOR gain values are in the normal range for all canals. In contrast, the results for a patient with idiopathic bilateral vestibular loss (Figure 3) shows absent eye velocity responses for every direction of head rotation, including all vertical canals. To correct for the total vestibular deficit, the patient made multiple overt catch-up saccades (red traces) at the end of the head rotation in every direction [10], [12].

**Figure 4:**
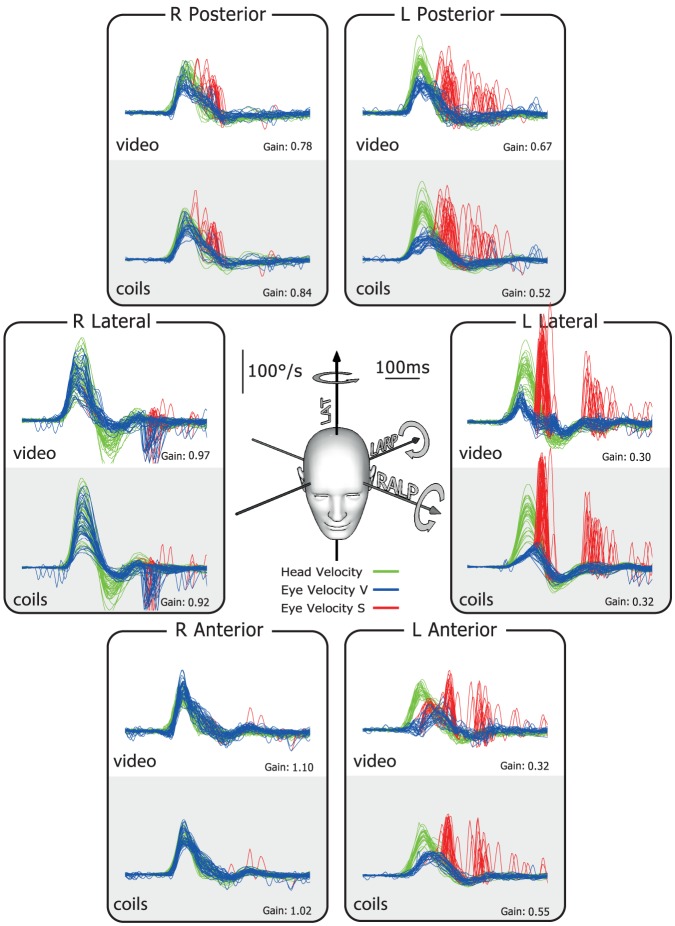
Simultaneous video and search coil head impulse recordings of all semicircular canals in a patient with left unilateral vestibular loss after surgery for vestibular Schwannoma. For every rotation direction activating canals on the healthy (right) side, the eye velocity response is around normal. However, for every rotation direction activating canals on the affected (left) side, there is a reduced or absent VOR response. In particular the vertical canals on the affected side have a clearly reduced function. To correct for the deficit on the affected left side, covert saccades appear during and overt saccades after head rotation (red traces).

The results for the patient with complete left unilateral vestibular loss after surgery for vestibular Schwannoma (Figure 4) show that eye velocity matches head velocity reasonably well for rightward head rotations (towards the healthy ear), but not for leftward head rotations (towards the affected ear). As is the case for the horizontal canal, reduced or absent slow-phase eye velocity appears during head turns towards the left anterior and left posterior canals. The slow-phase eye velocity is inadequate in response to stimulation of each canal on the affected side. The numerical VOR gain values show the clearly reduced gain for rotations towards the affected side. For the responses towards the affected ear, not only is the slow-phase eye velocity insufficient, but also there are many overt and covert saccades (red traces), which occur after the head impulses.

**Figure 5:**
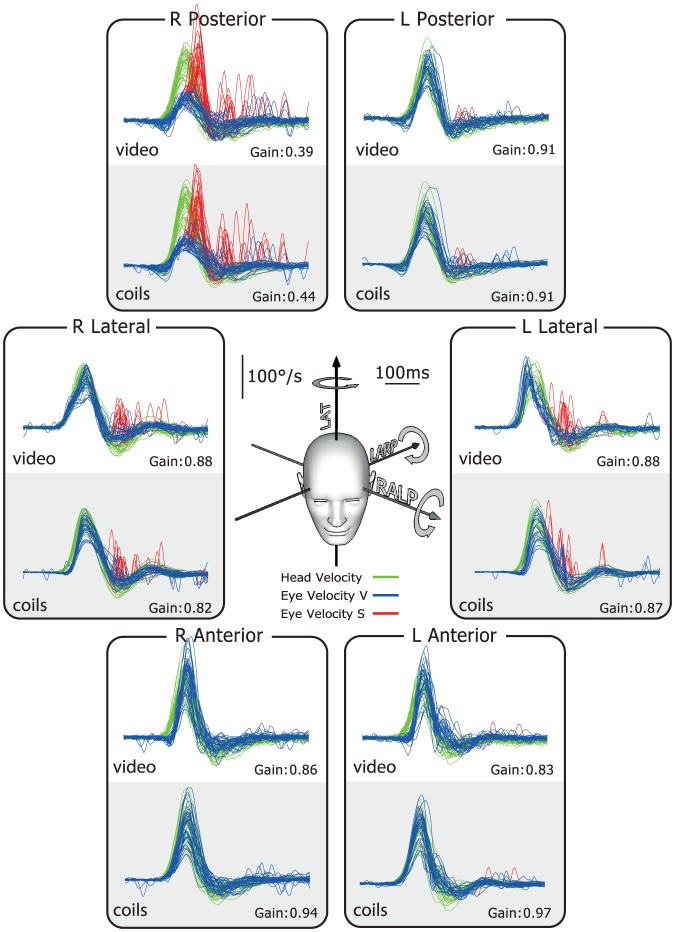
Simultaneous video and search coil head impulse recordings of all semicircular canals in a patient with single isolated loss of the right posterior semicircular canal. Results for a patient with a single isolated loss of the right posterior semicircular canal, as shown by prior testing using scleral search coils. For rotations, which would activate this single canal, there is a clear reduction of eye velocity (blue traces) during the head velocity stimulus (green traces), quickly followed by covert saccades (red traces). The responses for all other canals were in the normal range.

Figure 5 shows the results for a patient with an idiopathic isolated canal loss of the right posterior canal as identified by previous scleral search coil testing. The responses in all the canals were normal, with the exception of the single affected canal. Here the eye velocity response was not adequate and there were covert saccades (red traces) during the head impulses, for just the right posterior canal. Both scleral search coils and video correctly identified this patient's deficit in the posterior canal and clearly detected the corrective covert saccades (red traces). For both methods the VOR gain for this canal is significantly below normal (0.39 vHIT; 0.44 coils), whereas the VOR gains for all the other canals are in the normal range. The time series for a patient with bilateral posterior canal occlusion for intractable benign paroxysmal positional vertigo are provided in Figure S1.

**Figure 6:**
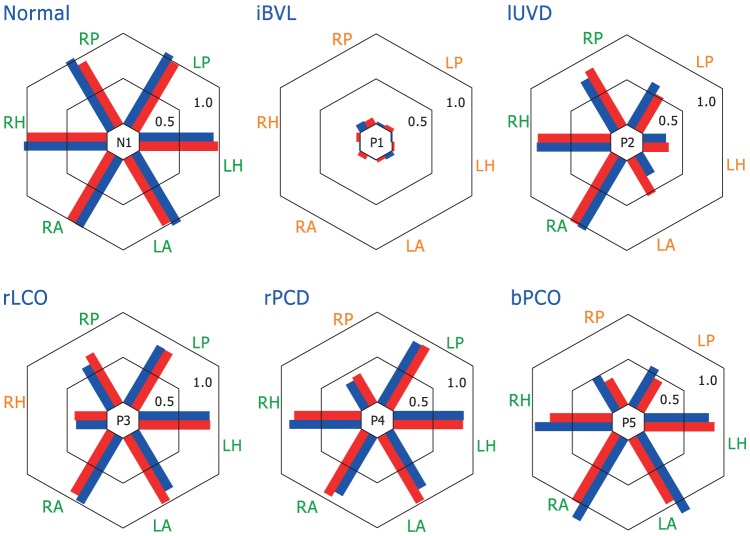
VOR gain comparison of simultaneous search coils and video measures. Bar plots of VOR gain for simultaneous search coils (red) and video measures (blue) for 6 representative subjects and patients. Bars are plotted side by side to facilitate comparison and arranged radially to indicate the results from head impulses delivered in planes of the: Right Anterior (RA), Left Anterior (LA), Right Horizontal (RH), Left Horizontal (LH), Right Posterior (RP), and Left Posterior (LP) semicircular canals. The data shown are for a range of patient conditions: Normal (same subject as in Figure 2); idiopathic Bilateral Vestibular Loss (iBVL; same patient as in Figure 3); left Unilateral Vestibular Deafferentation (lUVD; same patient as in Figure 4) after surgery for vestibular Schwannoma; right Lateral Canal Occlusion (rLCO) for intractable benign paroxysmal positional vertigo; idiopathic right Posterior Canal Dysfunction (rPCD; same patient as in Figure 5); and Bilateral Posterior Canal Occlusion (bPCO; same patient as Figure S1) for intractable benign paroxysmal positional vertigo. Results show a range of responses from canals in these patients, each with a pattern of canal responses that usually matches the expectation based on previous literature, but importantly the pattern of response on coils and video measures remains similar across a broad range of canal responses and diagnoses. (For individual VOR gain values, see Data S1.)

**Figure 7:**
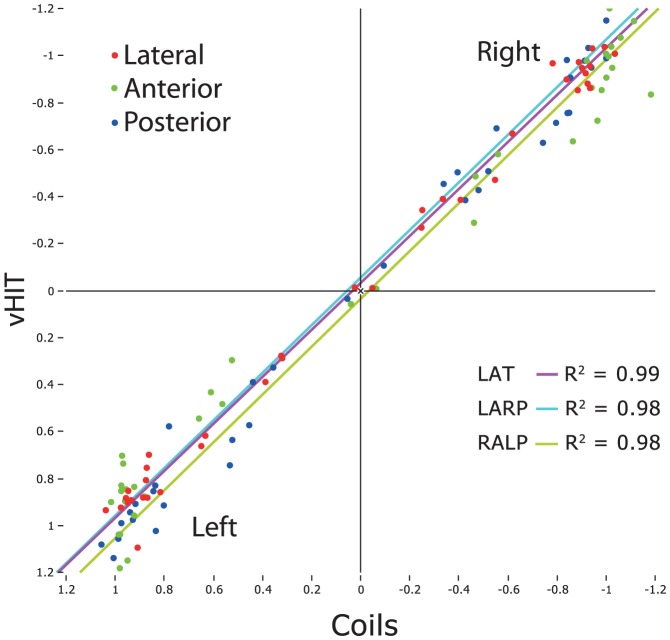
VOR regression plots from coils vs. video for lateral, anterior and posterior canals. The coefficients of determination (R^2^) for each regression are listed. In each case the correspondence is extremely strong as shown by both graphical data and statistical analysis.

The difference between average VOR gains for video vs. search coil across all subjects and patients was 0.01, n = 19 subjects×6 canals, SD = 0.11 (individual VOR gain values are provided as Data S1). Using a paired t-test t, this was not significantly different from zero, showing that VOR gains calculated with a new algorithm (see below) from vHIT measures are as accurate as those from search-coil measures. For LARP the mean difference between the two methods was 0.05 (n = 38 (19 subjects×2 canals), SD = 0.14) and for RALP the mean difference was −0.04 (*n* = 38, SD = 0.14). These were not statistically significantly different from zero. Figure 6 shows how closely the VOR gains for vHIT and search coils matched across all canals for a variety of different patient conditions (complete data set see also Figure S2). The coefficient of determination *R^2^* between VOR gain for vHIT and coils was 0.98 for the LARP plane, 0.98 for the RALP plane and 0.99 for the horizontal plane (Fig. 7).

### Gain calculation model and movement artifacts

In patients with low VOR gains, video recordings sometimes revealed a biphasic artifact at the beginning of the head impulse that was not apparent in the simultaneous search coil recordings (e.g. Figure 3, lateral canals). In contrast to search coil measures, which only detect angular rotations, video is also susceptible to linear movements. Therefore, we assumed that the video recording artifacts around peak head acceleration probably result from goggle slippage caused by movement of the skin relative to the skull when the head is passively rotated.

**Figure 8:**
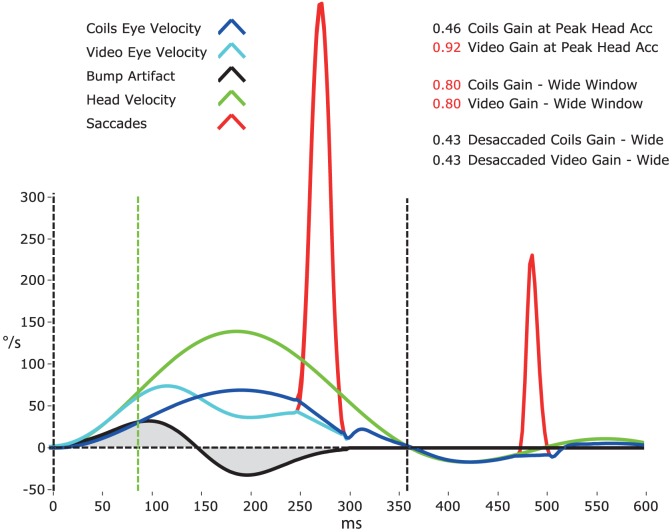
Head impulse gain calculation model. Gain calculation model for head impulses measured with video (cyan trace) and search coils (blue trace) taking into account movement artifacts from video goggle slippage (black trace) in the video signal. The video recording artifact around peak head acceleration (green dashed line) probably results from relative movement of the facial skin (on which the goggles ride) when the head is passively rotated (green trace). Since traditional VOR gain measurement over a narrow window, usually around peak head acceleration (green dashed line), is very sensitive to the influence of this artifact, we measured gain over a wide window from the beginning of the head impulse until the head velocity returns to 0°/s (black dashed lines). This gain calculation method is relatively unaffected by the biphasic movement artifact, because its positive component (manual acceleration of the head) and its negative component (deceleration) tend to cancel out (grey shaded areas) during the impulse. It is, however, susceptible to the influence of catch-up saccades (red trace), so eye velocity is desaccaded first. Gains calculated using this method are very similar for video and coils and quite comparable to the traditional gain measurement method for search coils around peak head acceleration.

Figure 8 demonstrates a gain calculation model for head impulses (green trace) measured with video (cyan trace) compared to search coils (blue trace). The model takes into account biphasic movement artifacts in the video signal from slippage of the video goggles (black trace). Because of the artifact, traditional VOR gain measurement at peak head acceleration (green dashed line) led to falsely high gains with video compared to search coils (model example: 0.92 vs. 0.46). Therefore, we measured gain over a wide window from the beginning of the head impulse until the head velocity returns to 0°/s (black dashed lines). Because the positive component (manual acceleration of the head) and the negative component (deceleration) of the biphasic movement artifact tended to cancel during the impulse (grey shaded areas), gain calculations for video and search coils were similar (0.80 both). However, covert saccades (red trace) during the head impulses led to falsely high VOR gain values for both methods. Therefore, the catch-up saccades were first detected based on an eye acceleration criterion and excluded from the analysis. Gains calculated with desaccaded eye velocity were very similar for video and search coils (0.43 both) and quite comparable to the traditional gain measurement method for search coils around peak head acceleration (0.46).

## Discussion

In this study we showed that the video head impulse test detects vertical canal dysfunction as well as the presently accepted gold standard - scleral search coils. Across the subjects and patients, although the recording techniques were very different, the results were very similar. Both recording methods identified the affected semicircular canals in patients and detected the resulting catch-up saccades.

In healthy subjects, both methods could demonstrate normal responses of the vestibulo-ocular reflex (VOR) for stimuli in all canal planes. Patients with bilateral loss of semicircular canal function showed loss of all vertical canal responses, as well as loss of horizontal canal responses. Patients with complete unilateral loss of vestibular function after surgery for vestibular Schwannoma showed reduced or absent responses for the affected anterior, posterior and horizontal canals, leaving the responses of the healthy vertical and horizontal canals little affected [13]–[15]. The patient with idiopathic loss of a single posterior canal illustrates that both methods were able to pinpoint the deficit of that particular canal by its reduced eye-velocity response.

This study shows that it is practical to measure not only the function of horizontal, but also of vertical semicircular canals with vHIT. To allow for two-dimensional eye movement measures based on video pupil tracking, we used a modified head impulse procedure for the vertical canals with eccentric gaze directed along the plane of head rotation, which elicits mainly vertical eye movements [7]. This way, we could dispense with complex video processing of torsional eye movements necessary for the analysis of traditional vertical head impulses with gaze directed straight ahead [14].

Vertical head impulses are more difficult to deliver than horizontal impulses, because the planes of the vertical canals lie diagonally in the head, and the neck movements along those diagonal planes are awkward to deliver and uncomfortable for the subject. This problem is minimized by using the modified technique described above with the head turned on trunk, but even so the range of movement for vertical head impulses is limited. In this mode of testing the eye is in an eccentric position, so the upper and lower eyelids restrict the vertical range and obtaining even illumination of the iris for successful pupil tracking is technically more demanding. Unfortunately, small eyes, especially in Asians, may sometimes hamper recording of vertical eye movements. Nevertheless, most of these problems can be overcome with practice by a skilled operator, so that the method is practical for clinical application in the majority of patients.

Compared to scleral search coils, vHIT is non-invasive and much easier to apply, but more susceptible to recording artifacts (Figure 8). However, these artifacts only become apparent in patients with reduced vestibular function (e.g. Figure 3, lateral canals), because they are otherwise concealed within the VOR response. The mostly biphasic artifacts are probably due to movement of the facial skin (and hence the goggles) relative to the head around peak head acceleration, even when using lightweight goggles with minimal slip. Unfortunately, the artifacts are located just at the spot where VOR gain has traditionally been measured [10]. To overcome this problem, we developed a new algorithm, which calculates gain during the entire head impulse, not just during a narrow time window around peak acceleration. This way the biphasic artifact cancels at least partially and interferes less with gain calculation.

### Clinical significance

This video method of recording eye movements during vertical head impulses provides evaluation of vertical semicircular canal function in patients in a clinical setting, even at the bedside, where scleral search coil recordings are impossible. The non-invasive and short (about 10–15 min) nature of vHIT also facilitates follow-up examinations to document recovery of patients e.g. after vestibular neuritis.

vHIT allows the physician to refine the clinical diagnosis and determine whether the entire vestibular nerve is affected, or just branches of it. Vestibular neuritis, for example, can affect the superior vestibular nerve, damaging the anterior and lateral canal, the inferior vestibular nerve, damaging only the posterior canal, or both [2]. So involvement of both the anterior and lateral canal confirms the diagnosis of ‘classic’ superior vestibular neuritis. Evidence of isolated loss of posterior canal function, on the other hand, confirms the diagnosis of inferior vestibular neuritis, differentiating this condition from a central vestibular disorder [16]. Furthermore, the method helps evaluating superior canal function in patients with superior canal dehiscence [17] or confirming posterior canal occlusion after surgery for intractable benign paroxysmal positional vertigo [6]. Combined with the vestibular evoked myogenic potential testing (VEMP) for otoliths, it means that the function of all vestibular sense organs can now be tested [18].

### Conclusion

vHIT detects dysfunction of individual vertical semicircular canals in vestibular patients. In contrast to search coils, which have been the only alternative to date, this new method of detecting dysfunction of individual vertical semicircular canals is quick, non-invasive and practical in clinics.

## Supporting Information

Data S1 **VOR gains for scleral search coil vs. video head impulse test across all normal subjects and patients (complementary to** **Figure 6** **and Figure S2).**
(XLSX)

Figure S1 **Bilateral surgical posterior canal occlusion for intractable benign paroxysmal positional vertigo.** Simultaneous video and search coil recordings of head impulse testing of all semicircular canals in a patient with bilateral surgical posterior canal occlusion for intractable benign paroxysmal positional vertigo. For every rotation direction activating the horizontal or anterior canals the eye velocity response is around normal. However, for rotations activating the posterior canals on both sides there is a reduced VOR response. To correct for the deficit on the affected posterior canals, overt saccades appear after head rotation (red traces).
(PDF)

Figure S2 **VOR gain comparison of simultaneous search coils and video measures.** Bar plots of VOR gain for simultaneous search-coil (red) and video measures (blue) for additional 6 subjects and 7 patients (complementary to Figure 6). Bars are plotted side by side to facilitate comparison and arranged radially to indicate the results from head impulses delivered in planes of the: Right Anterior (RA), Left Anterior (LA), Right Horizontal (RH), Left Horizontal (LH), Right Posterior (RP), and Left Posterior (LP) semicircular canals. Each bar shows the value of the VOR gain for lateral and vertical canals for subjects and patients. BVL = bilateral vestibular loss due to gentamicin vestibulotoxicity; uSCD = un-operated superior canal dehiscence; oSCD = operated superior canal dehiscence; rUVD = right unilateral vestibular deafferentation after surgery for vestibular Schwannoma; lUVD = left unilateral vestibular deafferentation after surgery for vestibular Schwannoma. The results show a range of responses from canals in these patients, each with a pattern of canal responses that usually matches the expectation based on previous literature, but importantly the pattern of response on coils and video measures remains similar across a broad range of canal responses and diagnoses. (For individual VOR gain values, see Data S1.)
(PDF)

Video S1 **Modified head impulse procedure for vertical semicircular canals.** Illustration of the head impulse examination procedure for individual semicircular canals in a patient with left unilateral vestibular deafferentation (lUVD). (A) Lateral (horizontal) head impulses. (B) Modified head impulses in the right anterior – left posterior (RALP) plane. (C) Modified head impulses in the left anterior – right posterior (LARP) plane. Note that for the modified vertical head impulses gaze is directed along the plane of head rotation to elicit vertical (instead of torsional) eye movements. Red: canal activation. Blue: canal inhibition. Green: canal dysfunction. Overt saccades that compensate for deficient canal function are indicated with click sounds. The movie has been created with the free iPhone or iPad app ‘aVOR’ developed by the first author [19].
(M4V)
