# Supplementary figures and images for: The Video Head Impulse Test (vHIT) Detects Vertical Semicircular Canal Dysfunction
Source: PLoS One. 2013 Apr 22;8(4):e61488. doi: 10.1371/journal.pone.0061488 (PMC3632590; doi:10.1371/journal.pone.0061488)

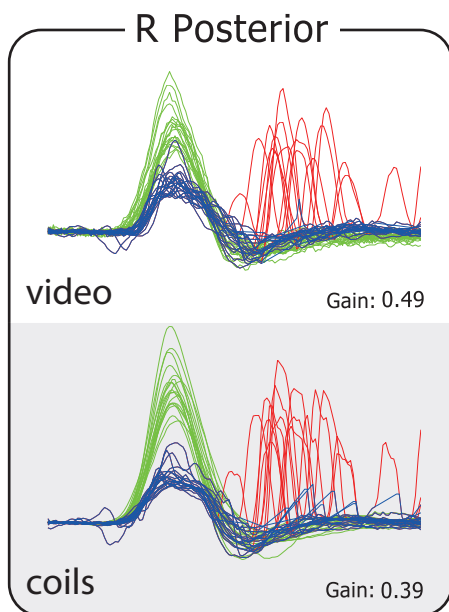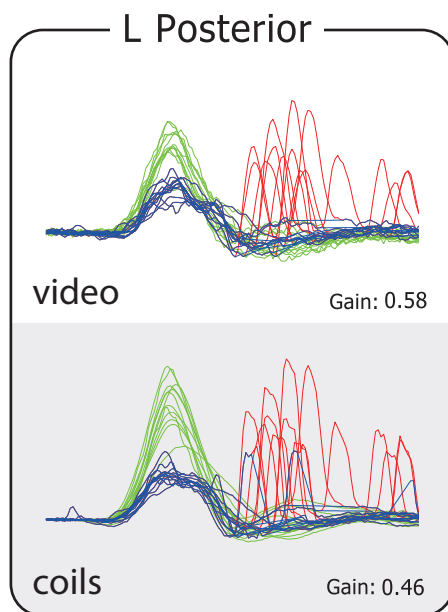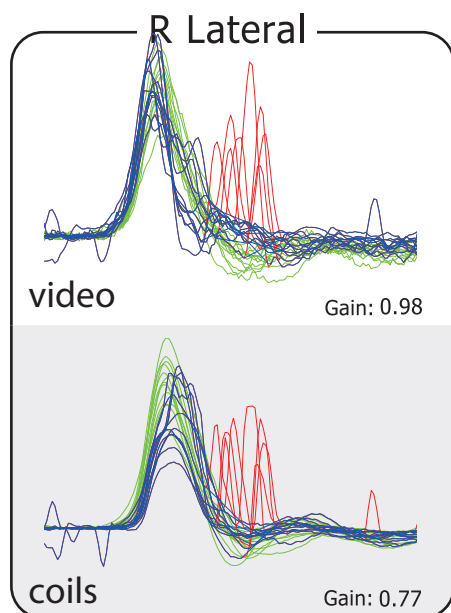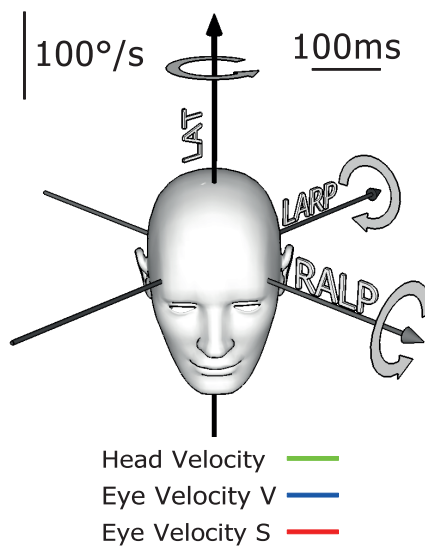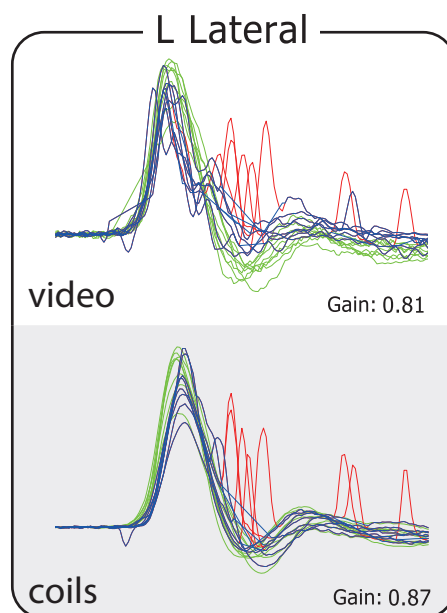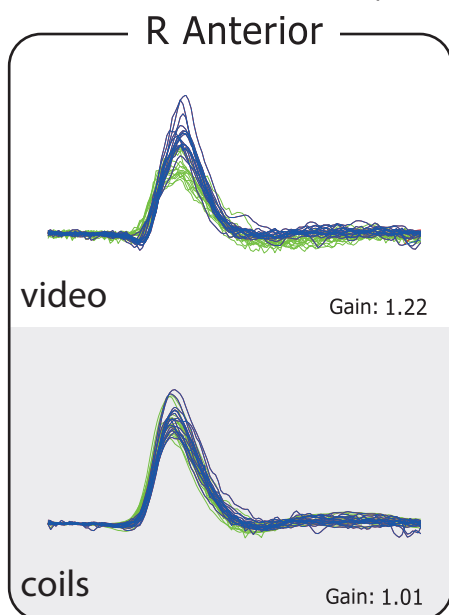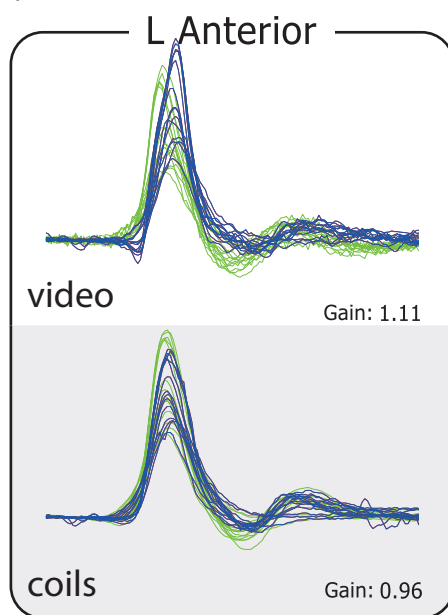

Supplement: Figure S1 — Bilateral surgical posterior canal occlusion for intractable benign paroxysmal positional vertigo. Simultaneous video and search coil recordings of head impulse testing of all semicircular canals in a patient with bilateral surgical posterior canal occlusion for intractable benign paroxysmal positional vertigo. For every rotation direction activating the horizontal or anterior canals the eye velocity response is around normal. However, for rotations activating the posterior canals on both sides there is a reduced VOR response. To correct for the deficit on the affected posterior canals, overt saccades appear after head rotation (red traces). (PDF) [file pone.0061488.s002.pdf]

## NORMALS

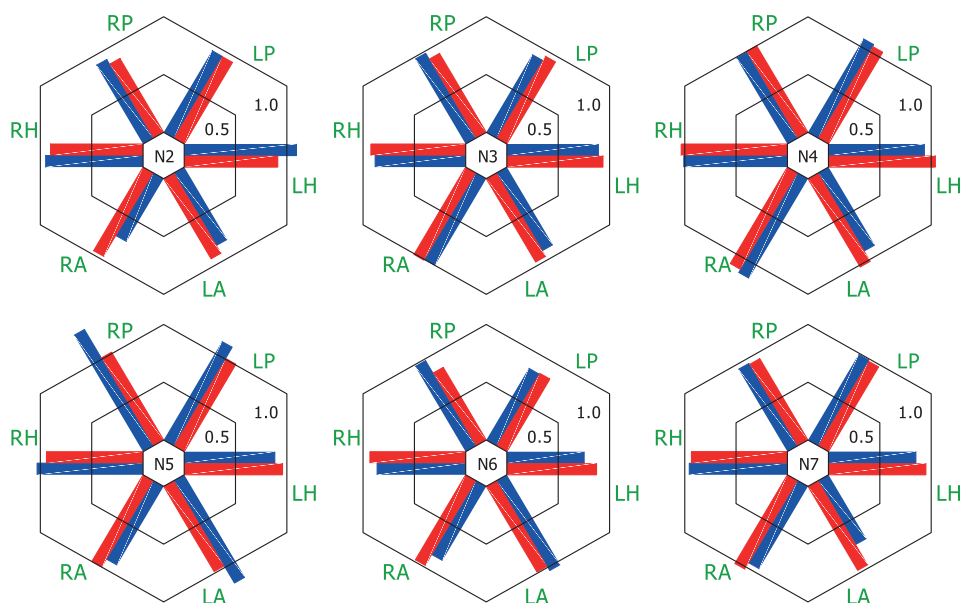

## PATIENTS

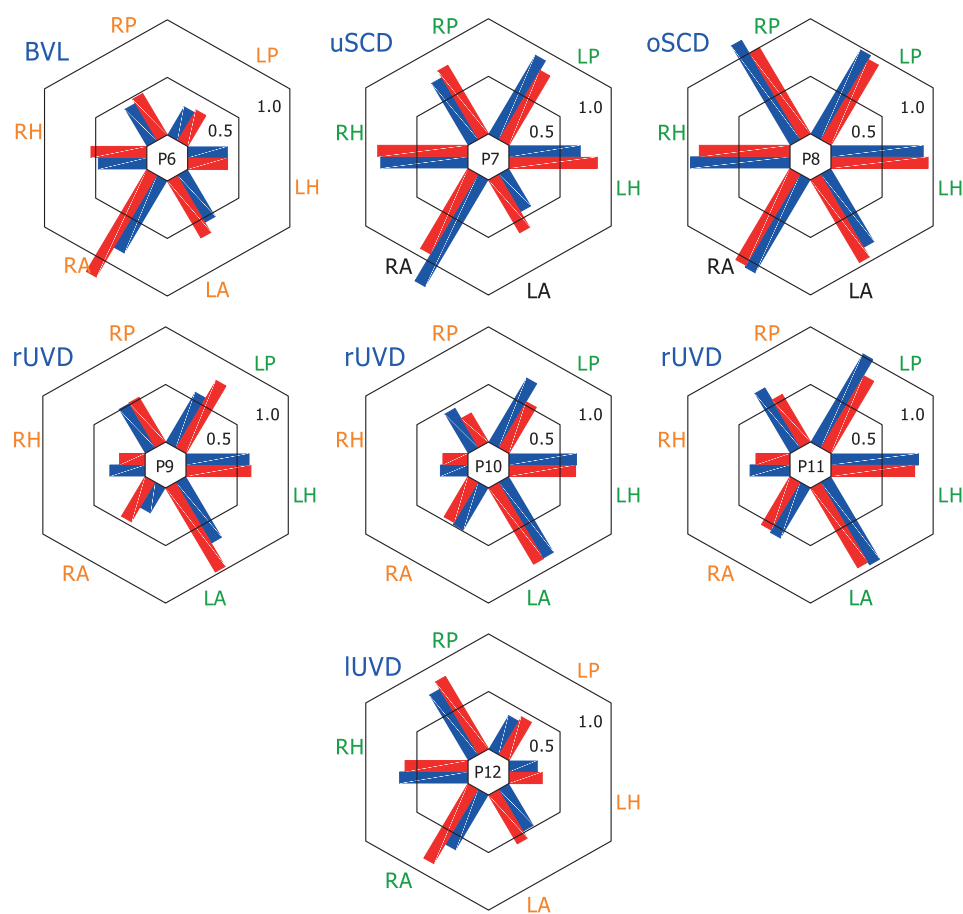

Supplement: Figure S2 — VOR gain comparison of simultaneous search coils and video measures. Bar plots of VOR gain for simultaneous search-coil (red) and video measures (blue) for additional 6 subjects and 7 patients (complementary to Figure 6). Bars are plotted side by side to facilitate comparison and arranged radially to indicate the results from head impulses delivered in planes of the: Right Anterior (RA), Left Anterior (LA), Right Horizontal (RH), Left Horizontal (LH), Right Posterior (RP), and Left Posterior (LP) semicircular canals. Each bar shows the value of the VOR gain for lateral and vertical canals for subjects and patients. BVL = bilateral vestibular loss due to gentamicin vestibulotoxicity; uSCD = un-operated superior canal dehiscence; oSCD = operated superior canal dehiscence; rUVD = right unilateral vestibular deafferentation after surgery for vestibular Schwannoma; lUVD = left unilateral vestibular deafferentation after surgery for vestibular Schwannoma. The results show a range of responses from canals in these patients, each with a pattern of canal responses that usually matches the expectation based on previous literature, but importantly the pattern of response on coils and video measures remains similar across a broad range of canal responses and diagnoses. (For individual VOR gain values, see Data S1.) (PDF) [file pone.0061488.s003.pdf]
